# *Helicobacter pylori vacA s1m1* genotype but not *cagA* or *babA2* increase the risk of ulcer and gastric cancer in patients from Southern Mexico

**DOI:** 10.1186/s13099-017-0167-z

**Published:** 2017-04-13

**Authors:** Adolfo Román-Román, Dinorah Nashely Martínez-Carrillo, Josefina Atrisco-Morales, Julio César Azúcar-Heziquio, Abner Saúl Cuevas-Caballero, Carlos Alberto Castañón-Sánchez, Roxana Reyes-Ríos, Reyes Betancourt-Linares, Salomón Reyes-Navarrete, Iván Cruz-del Carmen, Margarita Camorlinga-Ponce, Enoc Mariano Cortés-Malagón, Gloria Fernández-Tilapa

**Affiliations:** 1 Laboratorio de Investigación en Bacteriología, Facultad de Ciencias Químico Biológicas, Universidad Autónoma de Guerrero, Chilpancingo, Guerrero México; 2 Laboratorio de Investigación Clínica, Facultad de Ciencias Químico Biológicas, Universidad Autónoma de Guerrero, Avenida Lázaro Cárdenas S/N Ciudad Universitaria Sur, Col. La Haciendita, 39087 Chilpancingo, Guerrero México; 3 Hospital Regional de Alta Especialidad, Oaxaca, Oaxaca México; 4 Unidad Especializada en Gastroenterología Endoscopia, Chilpancingo, Guerrero México; 5 Servicio de Endoscopia, Instituto Estatal de Cancerología “Dr. Arturo Beltrán Ortega”, Acapulco, Guerrero México; 6 Servicio de Endoscopia, Hospital General “Dr. Raymundo Abarca Alarcón”, Chilpancingo, Guerrero México; 7 Unidad de Investigación Médica en Enfermedades Infecciosas y Parasitarias, Hospital de Pediatría, Centro Médico Nacional Siglo XXI, IMSS, Ciudad de México, México; 8 Laboratorio de Biología Molecular del Cáncer, Unidad de Investigación, Hospital Juárez de México, Ciudad de México, México

**Keywords:** *H. pylori*, Chronic gastritis, Gastric ulcer, Gastric cancer, *vacA*, *cagA*, *babA2*

## Abstract

**Background:**

The *vacA*, *cagA* and *babA2* genotypes of *Helicobacter pylori* are associated with gastric pathology. The objectives were to determine the frequency of infection and distribution of the *vacA*, *cagA* and *babA2* genotypes of *H. pylori* in patients with gastric ulcer, chronic gastritis and gastric cancer, and to evaluate the association of virulent genotypes with diagnosis.

**Methods:**

We studied 921 patients with symptoms of dyspepsia or with presumptive diagnosis of gastric cancer. The DNA of *H. pylori* and the *vacA*, *cagA* and *babA2* genes was detected by PCR in total DNA from gastric biopsies. The association of *H. pylori* and of its *cagA*, *vacA* and *babA2* genotypes with diagnosis was determined by calculating the odds ratio (OR).

**Results:**

Chronic gastritis was confirmed in 767 patients, gastric ulcer in 115 and cancer in 39. The prevalence of *H. pylori* was 47.8, 49.6 and 61.5% in those groups, respectively. *H. pylori* was more frequent in the surrounding tissue (69.2%) than in the tumor (53.8%). The *vacA s1m1* genotype predominated in the three groups (45.2, 61.4 and 83.3%, respectively). *H. pylori* was associated with cancer (OR_adjusted_ = 2.08; 95% CI 1.05–4.13; *p* = 0.035) but not with ulcer (OR_adjusted_ = 1.07; 95% CI 0.71–1.61; *p* = 0.728). The *s1m1* genotype was associated with ulcer and cancer (OR_adjusted_ = 2.02; 95% CI 1.12–3.62; *p* = 0.019 and OR_adjusted_ = 6.58; 95% CI 2.15–20.08; *p* = 0.001, respectively). *babA2* was associated with gastric cancer, and *cagA* was not associated with the diagnosis.

**Conclusions:**

In population from Southern Mexico, *H. pylori* and the *s1m1* genotype were associated with gastric cancer and the *s1m1*/*cagA*+/*babA2*+ strains predominated in tumor and adjacent tissue.

## Background

Persistent infection with *Helicobacter pylori* (*H. pylori*) induces chronic inflammation, tissue damage, deregulation of cellular regeneration and gastric carcinogenesis. The adhesion of *H. pylori* to epithelial cells of the gastric mucosa induces a marked inflammatory response, leading to chronic gastritis, peptic ulcer disease and gastric cancer [1, 2]. *H. pylori* colonize the gastric mucosa of up to 70 to 80% of the adults living in developing regions such as Africa and Latin America [3, 4]. In Mexico, the seroprevalence of *H. pylori* is 58 to 66.7% in people without symptoms of dyspepsia [5–8]; in patients with gastroduodenal pathology, the frequency of infection ranges from 60.1 to 87.4% [6, 9–12], a higher prevalence than that in some Southeast Asian countries [4]. However, not all carriers develop severe gastrointestinal diseases with clinical symptoms. Gastroduodenal diseases result from the interaction between genotypes of *H. pylori* and host and environment factors [13, 14].

The genomes of *H. pylori* are heterogeneous and encode different virulence factors that play an important role in the clinical outcome of the infection [1]. The proteins encoded by the *cagA*, *vacA* and *babA2* genes determine the pathogenicity of *H. pylori* and have been well described [15].

The *babA2* gene encodes the blood group antigen-binding adhesin (BabA), which binds to the fucosylated Lewis b antigen present on the surface of gastric epithelial cells. BabA facilitates colonization, persistence of infection and release of virulence factors of the bacterium. Infection with *babA2*-positive *H. pylori* has been associated with gastric ulcer, duodenal ulcer and gastric adenocarcinoma and is related to increased risk of severe disease when it coexists with the *cagA* gene and the *vacA s1* allele. Although three *bab* alleles have been identified (*babA1*, *babA2*, *babB*), only the product of the *babA2* gene is required for the binding of *H. pylori* to Lewis b. The association of BabA2 with severe gastric disease is controversial, but it is known that the interaction between BabA2 and Le^b^ activates the production of pro-inflammatory cytokines (CCL5, IL-8) and other molecules related to precancerous lesions (CDX2, MUC2) [1, 2, 15–18]. The frequency of *babA2*-positive *H. pylori* ranges from 21.7 to 82.3% in Latin American countries [10, 19, 20].

The cytotoxin-associated gene A (CagA) is a protein of 125–145 kDa encoded by the *cagA* gene, and an important virulence factor of *H. pylori*. The *cagA* gene is part of a genetic locus of 40 kb called *cag* pathogenicity island (*cag*-PAI) consisting of 27–31 genes, including those that encode a type IV secretion system (T4SS) that is responsible for the translocation of CagA to the cytoplasm of gastric epithelial cells by *cagA*-positive strains of *H. pylori* [21–23]. The *cagA* gene is a marker for the presence of *cag*-PAI; however, not all strains expressing the CagA protein genes express all *cag*-PAI genes. Based on the presence of *cagA*, the strains of *H. pylori* are grouped into *cagA*-positives and *cagA*-negatives. The prevalence of gastric diseases associated with *H. pylori* is higher among patients infected with *cagA*-positive strains. CagA is translocated into epithelial cells and activates signaling pathways that induce cellular changes and the production of IL-8 and other proinflammatory cytokines. The proinflammatory potential of *cagA*-positive *H. pylori* may explain its association with severe atrophic gastritis, peptic ulcer and gastric adenocarcinoma [24–26]. The frequency of *cagA*-positive *H. pylori* is 90–95% in Asian countries and 50–60% in Western countries. In Mexico, the prevalence of *cagA* varies from 47.6 to 63.4%, and the prevalence of anti-CagA+ antibodies among patients with gastric diseases reaches to 70.9% [12, 27]. The distribution of *cagA*-positive strains varies between regions and ethnic groups [28–30].

The vacuolating cytotoxin A (VacA) of *H. pylori* is associated with the risk of developing gastric cancer. VacA is encoded by the *vacA* gene, present in all strains of *H. pylori*. The *vacA* gene has a variable structure in the signal region (*s*), with *s1* or *s2* allele types; the intermediate region (*i*) exists as subtypes 1 and 2, while the middle region (*m*) has *m1* and *m2* allele types. The combination of allele types from each region results in the structure of the *vacA* gene, which determines the levels of toxin production. The *vacA s1*/*m1* strains of *H. pylori* produce high levels of cytotoxin; the *s1/m2* strains produce moderate levels, while the *s2*/*m2* strains produce minimal concentrations or do not produce it at all [31, 32]. The *s1m1* and *s1m2* genotypes generate VacA isoforms that cause direct damage to the gastric epithelium and stimulate an acute inflammatory process, which may lead to chronic gastritis or gastric ulcer [33–37]. The prevalence of the genotypes of *H. pylori* that express the most virulent factors changes with the geographic area [15], and the prevalence of infection with *H. pylori* *vacA s1m1* correlates with increased risk of disease [38].

The incidence of gastritis, ulcers and duodenitis has increased in the Mexican population in the last 10 years [39]. It is recognized that up to 80% of functional dyspepsia, 85–90% of peptic ulcers and 90% of gastric cancers are associated with infection by *H. pylori* [40]. The incidence rate of gastric cancer in Mexican men and women is 7.9 and 6.0/100,000, respectively [41]. However, despite the increase in the number of cases associated with *H. pylori*, there are few data on the prevalence of this infection in some gastroduodenal diseases, and still fewer on the distribution of the *vacA*, *cagA* or *vacA*/*cagA* genotypes in patients with peptic ulcers, non-ulcer dyspepsia or gastric cancer [6, 10, 11, 27, 42–45], while there is only one study on the frequency of the *vacA*, *cagA* and *babA2* genotypes in patients with chronic gastritis [10]. There are no studies on the prevalence of *H. pylori*, the distribution of the *vacA*, *cagA* and *babA2* genotypes and, simultaneously, on the relationship of these genes with clinical outcome in Southern Mexico population. The objective of this research was to determine the frequency of gastric infection and the distribution of the *vacA*, *cagA* and *babA2* genotypes of *H. pylori* in patients with gastric ulcer (GU), chronic gastritis (CG) and gastric cancer (GC). We also evaluated the association of these virulent genotypes with clinical outcome. This information will reveal the distribution of genotypes of *H. pylori* in Southern Mexico and may be useful for understanding the clinical relevance of genotyping in order to predict the clinical outcome of infection and to define therapeutic and prevention strategies for gastroduodenal diseases related to infection.

## Methods

### Patients

We studied 921 patients who were consecutively selected from those suffering from dyspepsia symptoms or who had presumptive diagnosis of gastric cancer. Eight hundred and eighty-two underwent upper endoscopy in the General Hospital Dr. Raymundo Abarca Alarcón or in the Specialized Unit for Gastroenterology Endoscopy in Chilpancingo; 39 underwent endoscopy for suspected gastric cancer in the State Institute of Oncology in Acapulco, Guerrero, Mexico. We included patients without antimicrobial treatment and without intake of proton pump inhibitors or of gastric pH neutralizers during the month prior to endoscopic procedure. Patients with immunosuppressant or nonsteroidal anti-inflammatory treatment were excluded from the study. The patients or their parents signed statements of informed consent. The patients were selected between March 2006 and May 2014. The project was approved by the Bioethics Committee of the Universidad Autónoma of Guerrero, by the Research Department of the State Cancer Institute and by the Department of Teaching and Research of the General Hospital Dr. Raymundo Abarca Alarcón.

### Endoscopy and histology

The endoscopy was performed after an overnight fast with a video processor and video gastroscope (Fujinon, Wayne, NJ USA). In patients with GC and GU, two biopsies were taken from the antrum, body or ulcer edge. In patients with GC, two biopsies were taken from the tumor and two from tissue adjacent to the cancer. A biopsy of each site was fixed immediately in formalin (10%) for histological examination and another was placed in a buffer solution (10 mM Tris pH 8.0, 20 mM EDTA pH 8.0, 0.5% SDS) for diagnosis of *H. pylori*. The biopsies intended for *H. pylori* detection were kept at −20 °C until processing. The biopsies fixed in formalin were embedded in paraffin. Tissue sections of 4 µm were stained with hematoxylin-eosin for histological study. The histopathological diagnosis was made according to the updated Sydney system [46], or based on the International Padova Classification of gastric dysplasia [47]. The endoscopic and histopathological findings were used only for diagnosis.

### Detection of *H. pylori* and genotyping of *vacA*

**Fig. 1:**
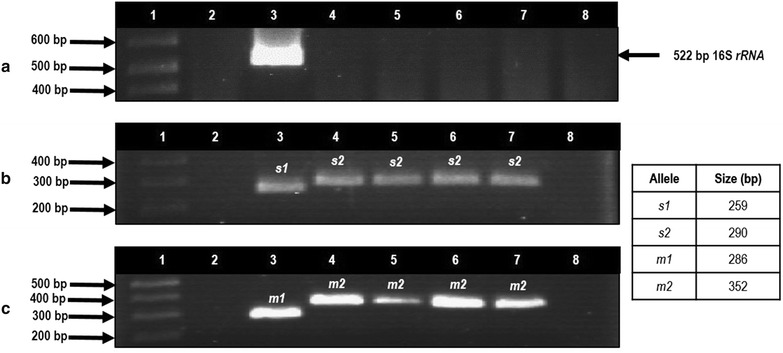
Oligonucleotides specificity used in the PCR for *16S rRNA* gene and *vacA s2, m2* alleles of *H. pylori.* **a** Amplification of 522 bp fragment of the 16S rRNA gene of *H. pylori*. *Lane 1* 1 kb plus molecular weight marker; *lane 2* negative control; *lane 3 H. pylori* strain ATCC43504; *lane 4 Staphylococcus aureus* strain; *lane 5 Escherichia coli* strain; *lane 6 Campylobacter sp* strain; *lanes 7 and 8* unidentified bacteria strains isolated from gastric biopsies. **b**, **c** PCR amplification products of *vacA* alleles, *s2* (**b**) and *m2* (**c**). *Lanes 3 H. pylori* strain 26695 (*vacA s1m1*); *lanes 4 H. pylori* strain 8822 (*vacA s2m2*); *lanes 5 H. pylori* strain Tx30 (*vacA s2m2*); *lanes 6* DNA of clinical strain HG-182 with *vacA s2m2* genotype isolated from a patient with antral chronic gastritis on March 10th, 2014 and *lanes 7 H. pylori vacA s2m2* in DNA from gastric biopsy UEGE-111 obtained from a patient with chronic gastritis on November 10th, 2007 (In this patient, the same genotype was detected in another biopsy taken on April 24th, 2007); *lbanes 8* **b** and** c**, empty

The total DNA from gastric biopsies and bacterial packets was extracted by the phenol-chloroform-isoamyl alcohol technique after proteinase K digestion [48]. The DNA of *H. pylori* was detected with conventional PCR using oligonucleotides directed to the *16S rRNA* gene [37]. PCR specificity was tested with DNA from different bacteria no *H. pylori*, isolated from gastric biopsies from the same patients. It was also tested with DNA from *Campylobacter sp*., a bacterium phylogenetically related to *H. pylori* (Fig. 1a). DNA integrity was verified adding a set of primers specific for *IL*-*1B* gene in the PCR for *H. pylori 16S rRNA* gene. *IL*-*1B* primers sequences were sense 5′-CAT TTG TCA GGT TCT TGA TC-3′ and antisense 5′-GAA GTT TAG TCT TCC CAC TT-3′ which amplified a 305 bp fragment (Fig. 3a–c). We only included in the study the samples *IL*-*1B*-positive. GC patients were considered *H. pylori*-positive when the *16S rRNA* gene was detected in the tumor, in the adjacent tissue, or both. The signal and middle regions of *vacA* were genotyped by PCR using the oligonucleotides previously used by Atherton et al., and Park et al, and according to the methodology described by Martínez-Carrillo et al, [6, 35, 49]. To ensure that we did genotyped correctly *vacA* alleles we tested by PCR a DNA sample from a *H. pylori* positive biopsy and DNA from a clinical strain of *H. pylori* that we isolated from a patient with chronic gastritis. We classified as genotype *s1m1* or *s2m2* by comparison with the amplicons obtained from strains ATCC43504 with genotyp*e s1m1* and 8822 (TX30) *vacA s2m2* of *H. pylori* (Fig. 1b, c).

### Detection of *cagA*

The positive samples for the *16S rRNA* gene of *H. pylori* were subjected to PCR for detection of *cagA* using the oligonucleotides *cagA*F 5′-ACAATGCTAAATTAGACAACTTGAGCGA-3′ and *cagA*R 5′ TTAGAATAATCAACAAACATCACGCCAT-3′ [50], which amplified a 297 bp fragment of the constant region; the set *cag*2F5′-GGAACCCTAGTCGGTAATG-3′ and *cag*4R 5′-ATCTTTGAGCTTGTCTATCG-3′ [51, 52] was used to amplify 500–850 bp of the 3′ variable region of *cagA*. The reaction mixture was prepared with 1.7 mM MgCl_2_, 0.2 mM dNTPs (Invitrogen, Carlsbad, CA, USA), 5 pmol of each oligonucleotide, 1 U of Taq DNA polymerase Platinum (Invitrogen, Carlsbad, CA, USA) and 300 ng of total DNA in a volume of 25 µL. The amplification program included one cycle at 94 °C for 5 min, 30 cycles at 94 °C for 40 s, 56 °C for 30 s and 72 °C for 50 s, and a final extension cycle at 72 °C for 10 min. The PCR products were subjected to electrophoresis on agarose gel (1.5%), the gels were stained with ethidium bromide and observed under ultraviolet light (UV). The samples were considered *cagA*-positive when at least one of the two bands was observed.

### Detection of *babA2*

**Fig. 2:**
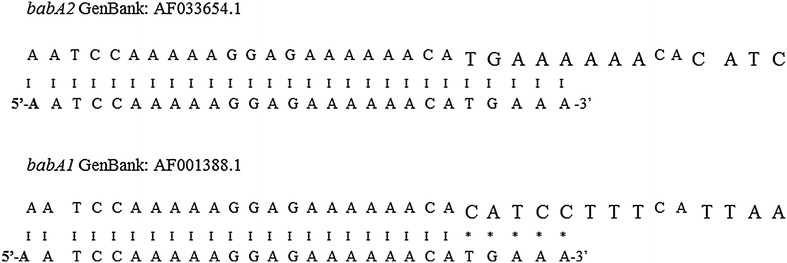
Forward primer binding to *babA2* and *babA1* gene sequences. Detection of *bab2A* gene is determined by perfect match of 3′- end from forward primer. Differences between *babA2* and *bab1* sequences are shown in the increased font size and matches are indicated by *vertical bars*; mismatches are indicated by *asterisks*. *babA1* and *babA2* have almost complete sequence homology, with the exception of an approximately 10 bp insert, found only in *babA2*, which creates a translational initiation codon in the signal peptide sequence. Gerhard et al. used this sequence difference to amplify the *babA2* gene selectively by mismatch PCR [16]

The presence of *babA2* was verified by mismatch PCR using the following oligonucleotides: F5´-AATCCAAAAAGGAGAAAAAACATGAAA-3′ and R5′-TGTTAGTGATTTCGGTGTAGGACA-3′, designed by Gerhard et al. [16], (Fig. 2). The amplification reaction was performed in a final volume of 15 μL, with 3.0 mM MgCl_2_, 0.25 mM dNTPs, 5 pmol of each oligonucleotide, 1 U of Taq DNA polymerase Platinum (Invitrogen, Carlsbad, CA, USA) and 600 ng of total DNA. The amplification program included an initial denaturation cycle at 95 °C for 3 min, 40 cycles at 95 °C for 30 s, 57 °C for 40 s, 72 °C for 45 s, and a final extension cycle at 72 °C for 5 min. The PCR products were subjected to electrophoresis on agarose gel (1.0%); the gels were stained with ethidium bromide and visualized under ultraviolet light (UV). The samples were considered *babA2*-positive when a band of 850 bp was observed.

DNA from the ATCC 43504 strain of *H. pylori* (*vacA s1m1*/*cagA*+/*babA2*+) was used as positive control in all PCR reactions; template DNA was substituted by sterile deionized water as negative control. DNA from a gastric biopsy was used as positive control for *s2* and *m2* allele types. All the PCR reactions were performed in a Mastercycler Ep gradient thermal cycler (Eppendorf, Germany).

### Statistical analysis

We used X^2^ or Fisher’s exact test to compare frequencies between groups, and analysis of variance (ANOVA) to compare means. The association of *H. pylori* and the *cagA*, *vacA* and *babA2* genotypes with the clinical outcome was determined by multinomial logistic regression models. A *p* value <0.05 was considered statistically significant. All statistics were calculated with Stata V.9.2 (College Station, Texas, USA).

## Results

### Patients and histological diagnosis

Of the 921 patients enrolled in the study, 83.3% had chronic gastritis, 12.5% had gastric ulcer and 4.2% had gastric cancer. The average age was 47.3 ± 16.2 years for cases of chronic gastritis (range 6–91 years); 54.9 ± 17.5 years for patients with gastric ulcer (range 9–90 years) and 59.2 ± 18.4 years for patients with gastric cancer (range 27–87 years). In chronic gastritis and gastric ulcer patients, the most frequent age group was 40–59 years (45.8% and 40.9%, respectively) and in the group of gastric cancer, 53% of patients were ≥60 years. Women predominated in all groups. The groups were significantly different in age (*p* < 0.001, ANOVA test), level of education and in living in overcrowded housing (*p* < 0.05) (Table 1).

**Table 1:** Sociodemographic characteristics of 921 Mexican patients with chronic gastritis, gastric ulcer and gastric cancer

	Diagnosis			
	CG n = 767 n (%)	GU n = 115 n (%)	GC n = 39 n (%)	*p* value
Age (years)				
≤20 years old	32 (4.2)	3 (2.6)	0	<0.001^δ^
20–39 years old	218 (28.4)	20 (17.4)	9 (23.1)	
40–59 years old	348 (45.4)	48 (41.7)	9 (23.1)	
≥60 years old	169 (22)	44 (38.3)	21 (53.8)	
Gender n (%)				
Female	471 (61.4)	65 (56.5)	24 (61.5)	0.603^Ф^
Male	296 (38.6)	50 (43.5)	15 (38.5)	
Education n (%)				
College or higher	367 (47.9)	43 (37.4)	3 (7.7)	<0.001^δ^
High school	114 (14.9)	16 (13.9)	5 (12.8)	
Junior high school	90 (11.7)	5 (4.4)	4 (10.3)	
Elementary school	143 (18.6)	31 (26.9)	15 (38.5)	
Uneducated	53 (6.9)	20 (17.4)	12 (30.7)	
Smoking habit n (%)				
No	441 (57.5)	53 (46.1)	20 (51.3)	0.060^Ф^
Current or previous smoker	326 (42.5)	62 (53.9)	19 (48.7)	
Alcohol drinking n (%)				
No	185 (24.1)	37 (32.2)	9 (23.1)	0.170^Ф^
Drinker or exdrinker	582 (75.9)	78 (67.8)	30 (76.9)	
Overcrowding n (%)				
No	477 (62.2)	90 (78.3)	25 (64.1)	0.004^Ф^
Yes	290 (37.8)	25 (21.7)	14 (35.9)	

*CG* chronic gastritis, *GU* gastric ulcer, *GC* gastric cancer

^Ф^ X^2^ test; ^δ^ Exact Fisher test

### Prevalence of *H. pylori*

**Fig. 3:**
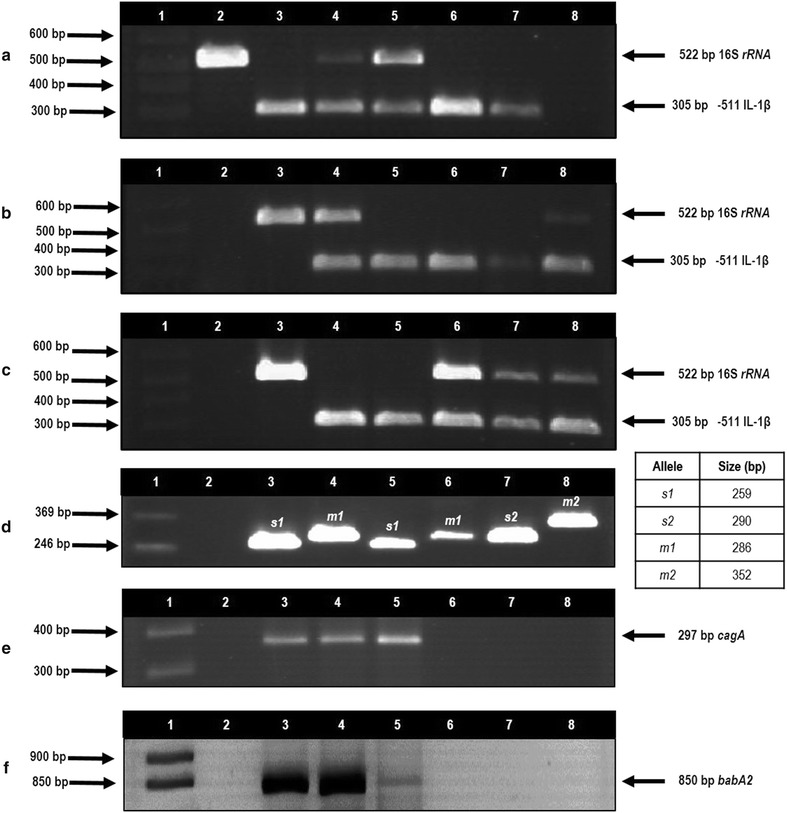
*H. pylori* detection in DNA from biopsies of patients with gastric pathology and genotyping of *vacA* and status of *cagA* and *babA2*. **a** PCR amplification product of *16S rR*NA gene in chronic gastritis patients. *Lane 1* 1 kb plus molecular weight marker; *lane 2* positive control (DNA from *H. pylori* 26695 strain); *lanes 3, 6, 7* negative samples; *lanes 4, 5* positive samples; *lane 8* negative control (without DNA). **b** PCR amplification product of *16S rRNA* gene in gastric ulcer patients. *Lane 1* 1 kb plus molecular weight marker; *lane 2* negative control (without DNA); *lane 3* positive control (DNA from *H. pylori* 26695 strain); *lanes 4, 8* positive samples; *lanes 5–7* negative samples. **c** PCR amplification product of *16S rRNA* gene in gastric cancer patients. *Lane 1* 1 kb plus molecular weight marker; *lane 2* negative control (without DNA); *lane 3* positive control (DNA from *H. pylori* 26695 strain); *lanes 4, 5* negative samples; *lanes 6–8* positive samples. **d** *vacA genotypes. Lane 1* plus molecular weight marker 123 bp; *lane 2* negative control (without DNA); *lanes 3, 4* positive control (DNA from *H. pylori* ATCC 43504 strain *vacA s1m1* genotype); *lanes 5, 6* DNA from gastric biopsy with *H. pylori vacA s1m1*; *lanes 7, 8* DNA from gastric biopsy with *H. pylori vacA s2m2*. **e** PCR amplification product of *cagA* gene. *Lane 1* 1 kb plus molecular weight marker; *lane 2* negative control (without DNA); *lane 3* positive control (DNA from *H. pylori* J99 strain *cagA*-positive); *lanes 4, 5* clinical samples with *H. pylori cagA*-positive, lanes 6-8 clinical samples *H. pylori cagA*-negative. **f** PCR amplification product of *babA2* gene. *Lane 1* 1 kb plus molecular weight marker; *lane 2* negative control (without DNA), *lane 3* positive control (DNA from *H. pylori* J99 strain *babA2*-positive); *lanes 4, 5* clinical samples *H. pylori babA2*-positive; *lanes 6–8* clinical samples *H. pylori babA2*-negative

The prevalence of *H. pylori* was 48.6% (448/921) (Fig. 3a–c), and the frequency of infection increased with disease severity (Table 2). *H. pylori* was more prevalent in patients with chronic gastritis and gastric ulcer in the range of 40–59 years (44.1 and 45.6%, respectively), but in gastric cancer, the highest frequency of *H. pylori*-positive patients (50%) were ≥60 years of age. Seventy-five percent (12/16) of patients younger than 20 years of age with chronic gastritis harbored *H. pylori cagA*+ in combination with different *vacA* genotypes. Seven of these patients were children aged 11–16 years old. A 19-year old patient with gastric ulcer was infected with a *vacA s1m1*/*cagA*+/*babA2*+ strain. The presence of *H. pylori* was investigated in DNA from tissue adjacent to cancer and from tumor in 13 of the 39 patients with gastric cancer; 53.8% (7/13) were *H. pylori*-positive in both sites and in 15.4% (2/13) the *16S rRNA* gene was detected in tissue adjacent to cancer but not in the tumor. The prevalence of *H. pylori* was not significantly different between groups (*p* = 0.243) (Table 2).

**Table 2:** *H. pylori* infection, status of *cagA/babA2* and *vacA* genotypes in patients with gastric pathology

	Diagnosis			*p* value
	CG n (%)	GU n (%)	GC n (%)	
*H. pylori*				
Negative	400 (52.2)	58 (50.4)	15 (38.5)	0.243^Ф^
Positive	367 (47.8)	57 (49.6)	24 (61.5)	
Total	767 (100)	115 (100)	39 (100)	
Distribution of *H. pylori* by age group				
≤20 years old	16 (4.4)	1 (1.8)	0	0.003^δ^
20–39 years old	120 (32.7)	10 (17.5)	4 (16.7)	
40–59 years old	162 (44.1)	26 (45.6)	8 (33.3)	
≥60 years old	69 (18.8)	20 (35.1)	12 (50)	
Total	367 (100)	57(100)	24 (100)	
*vacA* alleles				
*s1*	290 (83.3)	49 (84.5)	21(91.3)	
*s2*	58 (16.7)	9 (15.5)	2 (8.7)	
*m1*	201 (60.9)	36 (67.9)	20 (90.9)	
*m2*	129 (39.1)	17 (32.1)	2 (9.1)	
*vacA genotypes*				
*s1m1*	166 (45.2)	35 (61.4)	20 (83.3)	0.017^δ^
*s1m2*	62 (16.8)	7 (12.2)	0	
*s2m1*	9 (2.5)	0	0	
*s2m2*	41 (11.2)	9 (15.8)	2 (8.3)	
*s1m1/s1m2*	22 (6)	1 (1.8)	0	
*s2m1/s2m2*	4 (1.1)	0	0	
*s1m0*	18 (4.9)	5 (8.8)	1 (4.2)	
*s0m2*	4 (1.1)	0	0	
Non-typeable	41 (11.2)	0	1 (4.2)	
Total	367 (100)	57 (100)	24 (100)	
*cagA*				
Negative	158 (43.0)	22 (38.6)	10 (41.7)	0.925^Ф^
Positive	209 (57.0)	35 (61.4)	14 (58.3)	
Total	367 (100)	57 (100)	24 (100)	
*babA2*				
Negative	268 (73.0)	42 (73.7)	14 (58.3)	0.114^Ф^
Positive	99 (27.0)	15 (26.3)	10 (41.7)	
Total	367 (100)	57 (100)	24 (100)	
*vacA/cagA genotypes* ^a^				
*s2m2/cagA*−	26 (14.1)	3 (7.9)	1 (6.3)	0.114^δ^
*s2m2/cagA*+	7 (3.8)	2 (5.3)	0	
*s1m1/cagA*−	21 (11.4)	11 (28.9)	2 (12.5)	
*s1m1/cagA*+	130 (70.7)	22 (57.9)	13 (81.3)	
Total	184 (100)	38 (100)	16 (100)	

*CG* chronic gastritis, *GU* gastric ulcer, *GC* gastric cancer

^Ф^ X^2^ test, ^δ^ Exact Fisher test; s0: non-typeable for signal region. m0: non-typeable for middle region

^a^In this analysis only were included the infections caused by one *vacA* genotype

### *vacA* genotypes

The *vacA s1m1* genotype was the most frequent among *H. pylori*-positive patients, with 54.5% (244/448) (Fig. 3d). In 49.3% (221/448) of these patients, only the *s1m1* allele types were detected, while *s1m1* was found in co-infection with *s1m2* in 5.1% of patients (23/448). In 5.4% (24/448) of patients, the *s1* allele was detected but the *m* region was undetectable (*s1m0*); on the other hand, the *m2* allele was identified in 4 patients but the *s* region could not be identified (*s0m2*) (Table 2). *vacA s1m2* was found in 15.4% of samples (69/448). The same allele combinations of *vacA* were found in tissue adjacent to cancer and in tumor: 38.5% (5/13) *s1m1* and 15.4% (2/13) *s2m2* (Fig. 3d) (Table 3). It was impossible to genotype the *vacA* gene in 42 (9.4%) of the 448 *H. pylori*-positive patients. The prevalence of *vacA* genotypes and alleles varied with clinical outcome; *vacA s1m1* was the most frequent in all groups. Significant differences were found in the distribution of *vacA* genotypes between groups (*p* = 0.017) (Table 2).

**Table 3:** *H. pylori* and its virulence genes in tumor and adjacent tissue of patients with gastric cancer

Patient	Adjacent tissue n = 13					Tumor n = 13				
	*rRNA 16S H. pylori*	*vacA*		*cagA*	*babA2*	*rRNA 16S H. pylori*	*vacA*		*cagA*	*babA2*
		*s*	*m*				*s*	*m*		
IEC02	Negative					Negative				
IEC03	Positive	*s1*	*m1*	+	+	Positive	*s1*	*m1*	+	+
IEC04	Negative					Negative				
IEC05	Positive	*s1*	*m1*	+	–	Positive	*s1*	*m1*	+	–
IEC07	Negative					Negative				
IEC10	Positive	*s1*	*m1*	–	–	Negative	–	–	–	–
IEC11	Positive	*s2*	*m2*	–	+	Positive	*s2*	*m2*	–	+
IEC12	Positive	*s1*	*m1*	+	+	Negative	–	–	–	–
IEC16	Positive	*s1*	*m1*	+	+	Positive	*s1*	*m1*	+	+
IEC17	Positive	*s1*	*m1*	+	+	Positive	*s1*	*m1*	+	+
IEC19	Positive	*s2*	*m2*	–	–	Positive	*s2*	*m2*	–	–
IEC 20	Negative					Negative				
IEC 21	Positive	*s1*	*m1*	+	+	Positive	*s1*	*m1*	+	+
Total	13	*9*	*9*	6	6	13	*7*	*7*	5	5

The italic text refers to genes and alleles *s* or *m* of *vacA* of *H. pylori*

### Frequency of *cagA* and *babA2*

Four hundred and twelve of the 448 *H. pylori*-positive biopsies were tested for *cagA* (Fig. 3e). The *cagA* gene was detected in 62.6% (258/412) of patients studied; the frequency was similar among infected patients in the three groups. No significant differences were found in the frequency of *H. pylori*-*cagA*+ between groups (*p* = 0.925) (Table 2). In cancer, 71.4% (5/7) of tumor and surrounding tissue biopsies positive for *H. pylori* in both sites harbored *cagA*+ strains.

A total of 423 DNA samples were analyzed for *babA2* (Fig. 3f). The *babA2* gene was detected in 29.3% (124/423) of *H. pylori*-positive patients. *babA2* was found in patients from all groups but it was more frequent in gastric cancer; however, there were no significant differences in the frequency of *H. pylori*-*babA2*+ strains between groups (*p* = 0.114) (Table 2). Fifty-nine point seven percent (74/124) of the samples positive for *babA2*+ had also *vacAs1m1*/*cagA*+ . Seventy-one point 4 percent (5/7) of patients with infection in tumor and surrounding tissue were positive for *babA2*+ .

We analyzed the combination and the frequency of the most virulent genotypes *vacA*/*cagA*/*babA2*. The *vacA s1m1*/*cagA*+/*babA2*+ genotype was the most frequent in all groups, and its prevalence was higher in gastric cancer. The distribution of *vacA*/*cagA*/*babA2* genotypes was significantly different between groups (*p* = 0.041); data not shown. Of the patients with *H. pylori* in surrounding tissue and in tumor, 57.1% (4/7) harbored the allele combination *vacA s1m1*/*cagA*+/*babA2*+, 14.3% (1/7) harbored the *s2m2*/*cagA*−/*babA2*− genotype and 14.3% (1/7) the *s2m2*/*cagA*−/*babA2*+ genotype.

### Association of *H. pylori* and *vacA, cagA* and *babA2* genotypes with diagnosis

Infection with *H. pylori* was associated with gastric cancer (adjusted OR 2.08; 95% CI 1.05–4.13; *p* = 0.035) but not with gastric ulcer (adjusted OR 1.07; 95% CI 0.71–1.61; *p* = 0.728) (Table 4). A significant association was found between the *s1m1* genotype and ulcer and gastric cancer (OR_adjusted_ = 2.02; 95% CI 1.12–3.62; *p* = 0.019 and OR_adjusted_ = 6.58; 95% CI 2.15–20.08; *p* = 0.001, respectively). The *babA2* gene was associated with gastric cancer (OR_adjusted_ = 2.50; 95% CI 0.99–6.32; *p* = 0.052); *cagA* was not associated with clinical outcome (Table 4).

**Table 4:** Association of *H. pylori* and its virulence genes *vacA s1m1, cagA, babA2* with gastric ulcer and gastric cancer

	Diagnosis			
	Gastric ulcer		Gastric cancer	
	OR (CI 95%)	*p* value	OR^a^ (CI 95%)	*p* value
*H. pylori*				
Negative	1.0^b^		1.0^b^	
Positive	1.07 (0.71–1.61)	0.728	2.08 (1.05–4.13)	0.035
*vacA*				
*s2m2*	1.0^b^		1.0^b^	
*s1m1*	2.02 (1.12–3.62)	0.019	6.58 (2.15–20.08)	0.001
*cagA*				
Negative	1.0^b^		1.0^b^	
Positive	1.02 (0.56–1.86)	0.934	1.22 (0.47–3.17)	0.676
*babA2*				
Negative	1.0^b^		1.0^b^	
Positive	0.97 (0.50–1.85)	0.927	2.50 (0.99–6.32)	0.052
Genotype				
*s2m2/cagA*−	1.0^b^		1.0^b^	
*s2m2/cagA*+	2.5 (0.33–18.6)	0.374	–	–
*s1m1/cagA*−	4.3 (1.02–18.2)	0.047	1.8 (0.15–22.1)	0.639
*s1m1/cagA*+	1.5 (0.40–5.5)	0.550	2.1 (0.25–16.8)	0.502

^a^OR adjusted for age and overcrowding

^b^Reference group: chronic gastritis

## Discussion

*Helicobacter pylori* is an important human pathogen associated with most cases of peptic ulcer disease, gastritis and gastric adenocarcinoma. In most people, infection with *H. pylori* is restricted to the gastric antrum, but in some patients the infection spreads both through the body and antrum [53].

There are few studies on the prevalence of *H. pylori* and of its *vacA*, *cagA* and *babA2* genotypes in the Mexican population, and the data on the association of these genotypes with gastric diseases are still controversial in most countries. The clinical relevance and geographical distribution of the virulent genotypes of *H. pylori* is still a matter of debate. This study reports the prevalence and relationship of virulence genes (*vacA*, *cagA* and *babA2*) of *H. pylori* with clinical status in patients from South of Mexico.

The prevalence of *H. pylori* infection in chronic gastritis and gastric ulcer patients was 47.8% and 49.6%, respectively, lower than that reported in other studies [6, 10, 11]. An important finding of this study was that seven (1%) children aged 11-16 years had chronic gastritis and infection with *H. pylori cagA*+ , and that a 19-year-old was diagnosed with gastric ulcer and *H. pylori* *vacA s1m1*/*cagA*+/*babA2*+. Gonzalez-Valencia et al. also reported that children from 2 to 16 years with abdominal pain were infected with *s1* or *s2*/*cagA*+ genotypes. Infection with virulent genotypes of *H. pylori* at an early age may be related to the occurrence of gastric cancer before age 30. In gastric cancer patients, the prevalence of *H. pylori* was 61.5%, similar to that found in Mexican patients in a different geographical region (60%) [9] and exceeding that reported by other authors (38%) [44]. The differences in the prevalence of *H. pylori* in people from the same country may be due to the different number of biopsies analyzed for each patient, the variable number of bacteria harbored by the tissue studied, the difference in sensitivity and specificity of the PCR method used, the geographic region and the environmental health conditions of the population studied.

In gastric cancer patients, the frequency of *H. pylori* was higher in normal tissues adjacent to cancer (69.2%) than in the tumor (53.8%). Similar findings were made in Chinese patients [54] and in Mexican patients [44] with gastric cancer. Although *H. pylori* can survive in the tumor, the microenvironment of cancerous epithelium and the changes experienced by cancer cells are detrimental to the survival of the bacteria [54]. Zhang et al, even proposed that the atrophic mucosa and intestinal metaplasia are detrimental to the growth of *H. pylori,* and Tang et al, mention that *H. pylori* plays an important role in early gastric carcinogenesis, but that it probably has less influence on later stages of the disease [54, 55]. In this study, *H. pylori* is associated with gastric cancer but not with gastric ulcer.

*Helicobacter pylori* strains with the *s1* allele in the signal region of *vacA* were found in 83.3 and 84.5% of patients with chronic gastritis and gastric ulcer, respectively. The percentage increased to 91.3% in gastric cancer patients. With respect to the middle region, the *m1* allele was found in 60.9 and 67.9% of patients in the two groups without cancer, while *m1* strains were found in 90.9% of the patients with cancer. As has been demonstrated in other studies in the Mexican population [6, 10, 42, 43], the predominant allelic combination was *s1m1*, followed by *s1m2* in patients with GC, GU and CG. Our results show that 60% of *H. pylori*-positive patients were infected with virulent *vacA s1m1* strains, alone or in co-infection with the *s1m2* genotype. The *vacA s1m1* genotype was associated with GU and GC. The VacA protein, a product of the *s1m1* combination, induces a more severe infiltration of neutrophils, and has higher vacuolating and apoptosis-inducing activity than the *s2m2* variant. In addition, VacA inhibits the expansion of the T cells activated by bacterial antigens and thus helps *H. pylori* evade the adaptive immune response and promotes the persistence of infection [53–55]. These properties of VacA may explain the association of the *s1m1* isoform with gastric ulcer and cancer. Interestingly, we found infection with *H. pylori s2m2* in tumor and in tissue adjacent to cancer in two patients with gastric cancer; both strains were *cagA*-negative, but one was *babA2*-positive. Lopez-Vidal et al, also found the *s2* and *m2* alleles in Mexican patients with cancer [44]. This finding suggests that other virulence factors of *H. pylori* may be involved in cancer induction. It has been found that gastric cancer patients infected with *Tipα*+ strains of *H. pylori* produce significantly higher amounts of TNF-α than patients with chronic gastritis, and that the TNF-α-induced inflammatory response plays a significant role in the development of gastritis and gastric carcinoma associated with infection by *H. pylori* [56].

Although all strains of *H. pylori* contain the *vacA* gene, it was impossible to detect the *m* and *s* regions of this gene in the genomic DNA of 42 of the 448 patients infected. Similar results have been reported in the Mexican population [43, 44]. The genetic diversity of the *s* and *m* regions and the existence of undetectable *vacA* genes may explain the difficulty in genotyping some strains [45, 57, 58]. Moreover, *H. pylori* contain at least two copies of the *16S* and *23S rRNA* genes but only one of the *vacA* gene [59]. In some samples, the amplification signal of *16S rRNA* was almost undetectable (Fig. 3a, b); it is thus likely that the number of copies of the *vacA* gene was insufficient for detection by PCR.

The prevalence of *cagA* in this population was 57% in chronic gastritis patients, 61.4% in gastric ulcer patients and 58.3% in gastric cancer patients. This prevalence is lower than that reported in Central and South America [15, 19, 60], but it is in agreement with previous studies in Mexico [10, 27]. The *cagA*-positive strains of *H. pylori* have been associated with a more severe inflammation of the gastric mucosa that precedes atrophic gastritis, peptic ulcer and gastric cancer [61–65]. In this research, *cagA* was not associated with gastric ulcer or cancer. This finding is in agreement with those reported by other authors in Mexican patients [45]. It is likely that gastric ulcer and cancer are associated only with the CagA isoforms that contain repetitions of the EPIYA-C motif. The type and number of EPIYA motifs in CagA was not determined in this research.

Interestingly, *cagA* was found in 71.4% (5/7) of *H. pylori*-positive samples in tumor and surrounding tissue; the *s1m1*/*cagA*+/*babA2*+ genotype was found in 57.1% (4/7) and the *s1m1*/*cagA*+/*babA2*− genotype in 14.3% (1/7). This result is consistent with the activity of CagA to induce epithelial-mesenchymal transition and cell proliferation, inhibit apoptosis, promote the loss of tight junctions and carry out other functions related to tumor invasiveness and metastasis [53, 66]. The presence of the *s2m2*/*cagA*−/*babA2*− and *s2m2*/*cagA*−/*babA2*+ genotypes in tumor and surrounding tissue suggests that other bacterial compounds may be involved in the promotion of carcinogenesis and tumor maintenance.

The *babA2* gene was found only in 27, 26.3 and 41.7% of chronic gastritis, gastric ulcer and cancer patients, respectively, and was marginally associated with gastric cancer (OR_adjusted_ = 2.5, 95% CI 0.99–6.32, *p* = 0.052). The frequency of *babA2* in chronic gastritis patients was higher than that reported in Mexican patients [10], but lower than that reported for gastritis, gastric ulcer and cancer patients in other Central and South American countries [19, 20, 67, 68]. Oliveira et al, also found an association of *babA2* with gastric cancer in patients from Brazil [20]. It is likely that the association of *babA2* with more severe gastric diseases that was found in this study is related to its coexistence with *cagA* and *vacA s1m1* (59.7%), as suggested by Chen et al. [69].

## Conclusions

In conclusion, infection with *H. pylori* and related diseases occurred early in population of Southern Mexico. The prevalence of *H. pylori* was 47.8, 49.6 and 61.5% in chronic gastritis, gastric ulcer and cancer patients, respectively and the infection with this bacterium is associated with gastric cancer. The *s1* and *m1* alleles of *vacA* are predominant in this population, and the *s1m1* genotype is associated with gastric ulcer and cancer. The presence of the *s2m2*/*cagA*-negative genotype in gastric cancer patients suggests that other virulence factors of *H. pylori*, or other infectious agents, may be involved in the carcinogenic process. Additionally, some host factors may be interacting with the virulence factors of *H. pylori* and they may play an important role in the gastric carcinogenesis. The prevalence of *cagA* in South Mexico is lower than that found in other countries of Central and South America, and *cagA* was not associated with gastric ulcer or cancer. The *s1m1*/*cagA*+/*babA2*+ strains of *H. pylori* predominated in the tumor and in the surrounding tissue, and their presence may be related to the likelihood of invasion and metastasis.
